# Melatonin confers heavy metal-induced tolerance by alleviating oxidative stress and reducing the heavy metal accumulation in *Exophiala pisciphila*, a dark septate endophyte (DSE)

**DOI:** 10.1186/s12866-021-02098-1

**Published:** 2021-02-05

**Authors:** Yang Yu, Zhaowei Teng, Zongmin Mou, Yan Lv, Tao Li, Suiyun Chen, Dake Zhao, Zhiwei Zhao

**Affiliations:** 1 State Key Laboratory for Conservation and Utilization of Bio-resources in Yunnan, Yunnan University, Kunming, China; 2 Department of Orthopedics, The Sixth Affiliated Hospital of Kunming Medical University, Yuxi, Yunan China; 3 Department of Orthopedics, The First people’s Hospital of Yunnan Province, Kunming, China; 4 Biocontrol Engineering Research Center of Plant Disease and Pest, Yunnan University, Kunming, China; 5 Biocontrol Engineering Research Center of Crop Disease and Pest, Yunnan University, Kunming, China; 6 School of Ecology and Environmental Science, Yunnan University, Kunming, China

**Keywords:** Dark septate endophytes, Heavy metals accumulation, Melatonin, Biosynthesis genes, Oxidative stress

## Abstract

**Background:**

Melatonin (MT), ubiquitous in almost all organisms, functions as a free radical scavenger. Despite several reports on its role as an antioxidant in animals, plants, and some microorganisms, extensive studies in filamentous fungi are limited. Based upon the role of melatonin as an antioxidant, we investigated its role in heavy metal-induced stress tolerance in *Exophiala pisciphila*, a dark septate endophyte (DSE), by studying the underlying mechanisms in alleviating oxidative stress and reducing heavy metal accumulation.

**Results:**

A significant decrease in malondialdehyde (MDA) and oxygen free radical (OFR) in *E*. *pisciphila* was recorded under Cd, Zn, and Pb stresses as compared to the control. Pretreatment of *E*. *pisciphila* with 200.0 μM exogenous melatonin significantly increased the activity of superoxide dismutase (SOD) under Zn and Pb stresses. Pretreatment with 200.0 μM melatonin also lowered Cd, Zn, and Pb concentrations significantly. Melatonin production was enhanced by Cd, Cu, and Zn after 2 d, and melatonin biosynthetic enzyme genes, *E*. *pisciphila* tryptophan decarboxylase (*EpTDC1*) and serotonin *N*-acetyltransferase (*EpSNAT1*), were transcriptionally upregulated. The overexpression of *EpTDC1* and *N*-acetylserotonin *O*-methyltransferase (*EpASMT1*) in *Escherichia coli* and *Arabidopsis thaliana* enhanced its heavy metal-induced stress tolerance. The overexpression of *EpTDC1* and *EpASMT1* reduced the Cd accumulation in the whole *A. thaliana* plants, especially in the roots.

**Conclusions:**

Melatonin conferred heavy metal-induced stress tolerance by alleviating oxidative stress, activating antioxidant enzyme SOD, and reducing heavy metal accumulation in *E. pisciphila*. Melatonin biosynthetic enzyme genes of *E. pisciphila* also played key roles in limiting excessive heavy metal accumulation in *A. thaliana*. These findings can be extended to understand the role of melatonin in other DSEs associated with economically important plants and help develop new strategies in sustainable agriculture practice where plants can grow in soils contaminated with heavy metals.

**Supplementary Information:**

The online version contains supplementary material available at 10.1186/s12866-021-02098-1.

## Background

Melatonin (MT)-an ancient indole molecule-dates back to 3.5 billion years ago, and occurs in almost all organisms including animals, plants, and microorganisms [1–3]. It functions as a free radical scavenger, scavenging the excess reactive oxygen species (ROS) and reactive nitrogen species (RNS) produced in respiration and photosynthesis [4, 5]. Under heavy metal stress conditions, the levels of ROS and RNS increase significantly, causing serious oxidative stress damage to the organism [6, 7]. Reports show an increase in melatonin levels by various heavy metals such as cadmium (Cd), vanadium (V), and Zinc (Zn) treatment in plants [7–9]. Melatonin production is promoted by upregulating the expression of biosynthesis enzyme genes containing tryptophan decarboxylase (TDC), tryptophan hydroxylase (TPH), serotonin *N*-acetyltransferase (SNAT), and *N*-acetylserotonin *O*-methyltransferase (ASMT), and then enhanced heavy metal stress resistance for plants [3, 9–12]. Elevated melatonin levels possibly help improve the heavy metal-induced stress tolerance by scavenging excess ROS and RNS.

Although the role of melatonin as an antioxidant conferring heavy metal-induced stress tolerance is widely studied in animals, plants, and some microorganisms, its role in filamentous fungi lacks extensive studies [13]. Since melatonin originated from the primitive bacteria (cyanobacteria and α-proteobacteria) and has existed throughout the evolution of all organisms [14, 15], the role of filamentous fungi in the melatonin evolution process cannot be overlooked [5].

Dark septate endophytes (DSEs), a group of dark pigmented and septate hyphae fungi, widely colonize in the plants’ roots and play an important role in plant growth and nutrient uptake [16]. Notably, these fungi wildly inhabit in most roots of plants that grow in mine smelting region with high concentrations of plumbum (Pb), Zn, and Cd [17, 18]. We asked whether DSEs played any role in the growth of plants in soils with high concentrations of heavy metals. We isolated a highly heavy metal tolerant DSE strain, *Exophiala pisciphila*, from the roots of *Arundinella bengalensis* growing in an old mine smelting site in Yunnan Province, Southwest China. Based on the report that the Pb, Cd, and Zn concentrations in the dry weight of *E*. *pisciphila* hyphae reach over 25, 4.9, and 16.0% respectively [16], we assumed that its survival in the soil with high concentrations of heavy metals was due to its heavy metal-induced stress tolerance. Based on the role of melatonin as an antioxidant, we hypothesized that it possibly participates in heavy metal-induced stress tolerance in *E. pisciphila*. To test our hypothesis, we investigated its role in heavy metal-induced stress tolerance by studying the underlying mechanisms in alleviating oxidative stress and reducing heavy metal accumulation, and the expression of genes controlling melatonin biosynthesis.

## Results

### Exogenous melatonin alleviated heavy metal-induced oxidative stress in *Exophiala pisciphila*

**Fig. 1:**
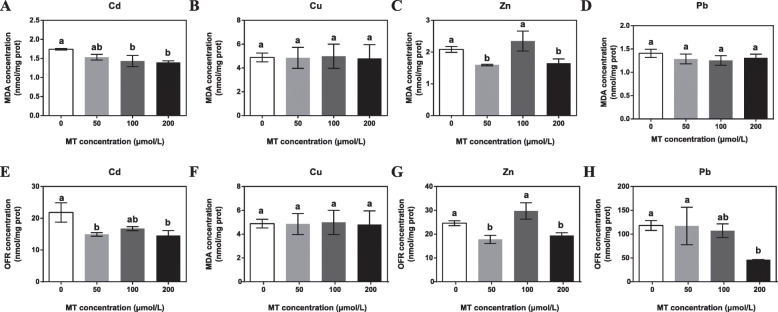
The effects of melatonin (MT) on heavy-metal induced oxidative stress in *Exophiala pisciphila*. *E. pisciphila* were pretreated with 0, 50.0, 100.0, or 200.0 μM MT for 1 d and then subjected to 111.2 mg L^− 1^ Cd^2+^ (A, E), 100.0 mg L^− 1^ Cu^2+^ (B, F), 1010.0 mg L^− 1^ Zn^2+^ (C, G), or 800.0 mg L^− 1^ Pb^2+^ (D, H) stress for 2 d. The MDA (A-D) and OFR (E-H) concentrations in *E*. *pisciphila* were measured. Data are means ± SD (*n* = 4). Columns with different letters denote significant difference at *P* < 0.05 according to Duncan’s multiple range test

Oxidative stress, one of the most serious damages in organisms by heavy metals [1], results in a significant increase in the levels of ROS and RNS. Since melatonin is a natural scavenger of ROS and RNS, we investigated the effect of exogenous melatonin on oxidative stress in *E*. *pisciphila* by assessing the increases in malondialdehyde (MDA) and oxygen free radical (OFR) concentrations. The MDA concentration of the isolate gradually decreased as the exogenous melatonin concentration application increased under Cd, Zn, and Pb stresses (Fig. 1a and c). The MDA concentration of the isolate under Cd and Zn stresses is significantly lower than the control (Figs. 1a and c). In contrast, isolates treated with melatonin under Cu and Pb stress did not exhibit any notable difference in MDA concentration as compared to the control (Fig. 1b and d).

We recorded a significant decrease in the OFR concentration in isolates pretreated with exogenous melatonin under Cd, Zn, and Pb stresses as compared to the control (Figs. 1e, g, and h). The pretreatment with 200.0 μM exogenous melatonin significantly reduced 33.31, 27.64, and 60.91% of OFR under Cd, Zn, and Pb stress respectively (Fig. 1e, g, and h). In contrast, Cu stress does not show any significant difference in OFR concentration (Fig. 1f). The results indicated that pretreatment with exogenous melatonin relieved the oxidative stress caused by Cd, Zn, and Pb in *E*. *pisciphila*.

### Exogenous melatonin enhanced superoxide dismutase (SOD) activity and reduced heavy metal accumulation in *Exophiala pisciphila*

**Fig. 2:**
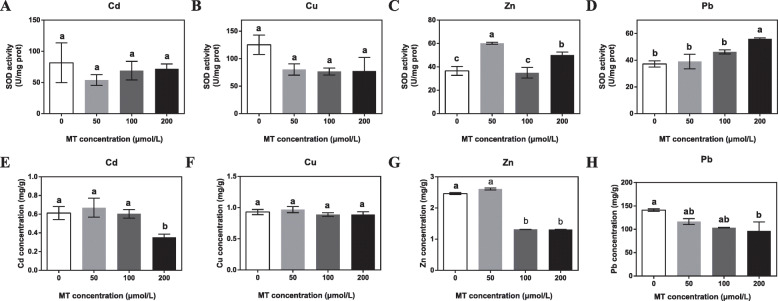
The effects of exogenous melatonin (MT) on superoxide dismutase (SOD) activity and decreased heavy metal accumulation in *Exophiala pisciphila*. *E. pisciphila* were pretreated with 0, 50.0, 100.0, or 200.0 μM melatonin (MT) for 1 d and then subjected to 111.2 mg L^− 1^ Cd^2+^ (A, E), 100.0 mg L^− 1^ Cu^2+^ (B, F), 1010.0 mg L^− 1^, Zn^2+^ (C, G), or 800.0 mg L^− 1^ Pb^2+^ (D, H) stress for 2 d. The SOD (A-D) activity and heavy metal (E-H) content of *E*. *pisciphila* were measured. Data are means ± SD (*n* = 4). Columns with different letters denote significant differences at *P* < 0.05 according to Duncan’s multiple range test

Since our results indicated that melatonin alleviated oxidative stress, we decided to examine its effect on SOD, one of the antioxidant enzymes. Pretreatment of *E. pisciphila* with 200.0 μM exogenous melatonin showed no notable difference was observed in the SOD activity under Cd and Cu stresses (Fig. 2a and b). In contrast, significantly increased the activity of SOD by 30.01 and 33.45% under Zn and Pb stresses respectively, as compared to the control (Fig. 2c and d).

Preventing excessive heavy metal accumulation is an important way to limit the deleterious impact on organisms [1]. We explored whether pretreatment of *E. pisciphila* with exogenous melatonin affected the Cd, Cu, Zn, and Pb concentrations. Figure 2e-h show that an increase in melatonin concentration gradually reduces the heavy metal concentration, and Cd, Zn, and Pb concentrations when pretreated with 200.0 μM melatonin are significantly reduced by 32.24, 46.7, and 31.55% respectively. The results indicate that exogenous melatonin promoted heavy metal tolerance by enhancing SOD activity and reducing heavy metals accumulation.

### *EpTDC1*, *EpSNAT1*, and *EpASMT1* differed from that in plants and animals

**Fig. 3:**
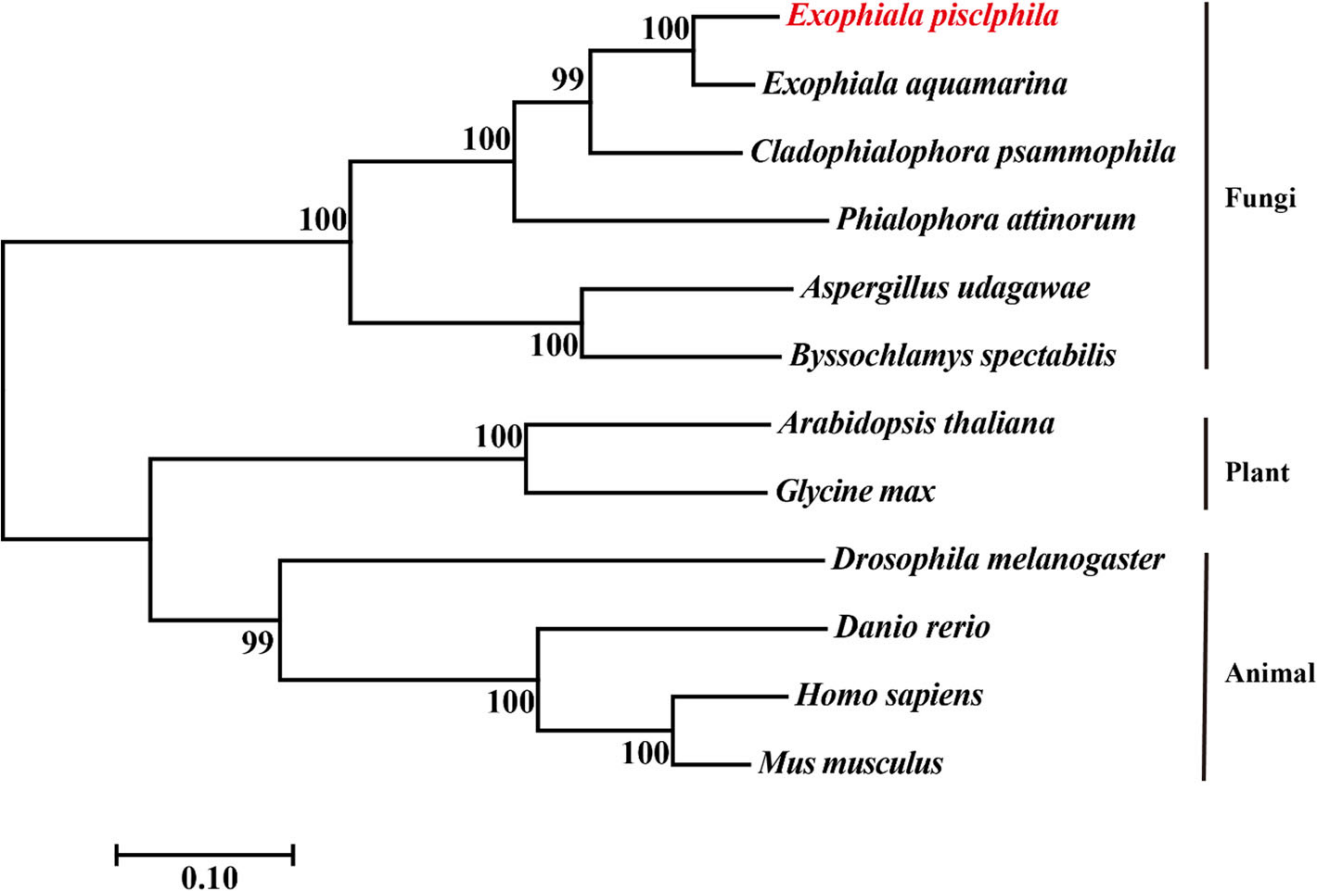
A phylogenetic tree of tryptophan decarboxylases (TDCs) based on amino acid sequences

Based upon the transcriptome data of *E*. *pisciphila* [19], cDNA sequences (*EpTDC1*, *EpSNAT1*, and *EpASMT1*) of three melatonin biosynthetic enzymes were obtained. The subcellular localization of proteins EpTDC1, EpSNAT1, and EpASMT1 were predicted on the cytoplasm, cytoplasm, and nucleus respectively (Table S1). Amino acid BLAST search revealed the presence of the EpTDC1, EpSNAT1, and EpASMT1 homologs in various animals and plants. The phylogenetic tree indicated that EpTDC1, EpSNAT1, and EpASMT1 were clustered together in fungi, which formed a separate clade from animals and plants (Fig. 3; Supplementary Fig. S1 and S2).

### Heavy metals stresses-induced upregulated *EpTDC1* and *EpSNAT1*, and downregulated *EpASMT1* expression, and promoted melatonin biosynthesis in *Exophiala pisciphila*

**Fig. 4:**
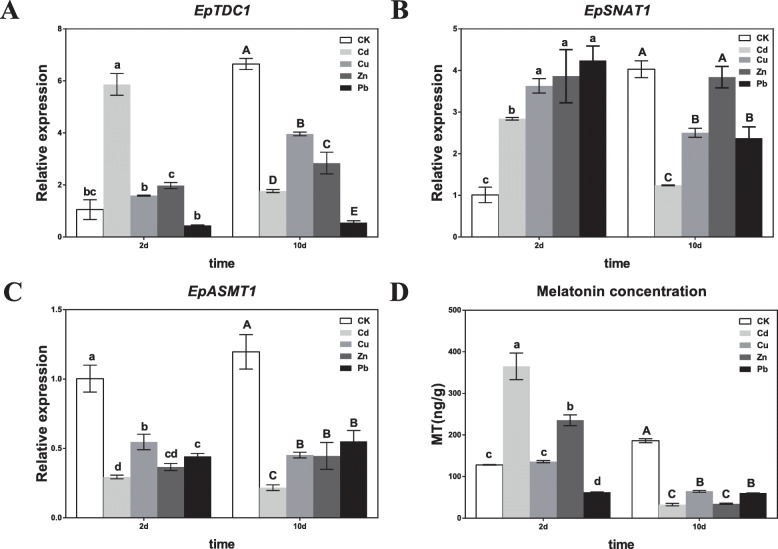
Heavy metal enhanced the expression of *EpTDC1*, *EpSNAT1*, *EpASMT1*, and the accumulation of melatonin in *E. pisciphila*. The isolates were pretreated with 111.2 mg L^− 1^ Cd^2+^, 100.0 mg L^− 1^ Cu^2+^, 1010.0 mg L^− 1^ Zn^2+^, and 800.0 mg L^− 1^ Pb^2+^ respectively for 2 or 10 d. The relative expression of *EpTDC1* (A), *EpSNAT1* (B), and *EpASMT1* (C) was analyzed by real-time RT-PCR, and the melatonin concentrations (D) were detected by HPLC. Data are means ± SD (*n* = 3). Columns with different letters denote significant differences at *P* < 0.05 according to Duncan’s multiple range test

Based on our results that exogenous melatonin promoted heavy metal tolerance by enhancing SOD activity and reducing heavy metal accumulation, we further investigated the effects of heavy metal on melatonin biosynthesis. We measured melatonin concentrations by high-performance liquid chromatography (HPLC) and the relative expression of *EpTDC1*, *EpSNAT1*, and *EpASMT1* by real-time RT-PCR in *E. pisciphila*. During the early treatment period (2 d), Cd, Cu, and Zn induced melatonin production (Fig. 4d). Simultaneously, expressions of *EpTDC1* (Fig. 4a) and *EpSNAT1* (Fig. 4b) was transcriptionally upregulated, and *EpASMT1* (Fig. 4c) was downregulated compared to the control. The expression of *EpTDC1*, *EpSNAT1*, and *EpASMT1,* and melatonin concentration lowered compared to the control after 10 d (Fig. 4a-d). The results indicated that heavy metals rapidly induced melatonin biosynthesis after 2 d that was clearly correlated to the upregulation of *EpTDC1* and *EpSNAT1* expression.

### *EpTDC1* and *EpASMT1* conferred heavy metals-induced stress tolerance in *Escherichia coli* and *Arabidopsis thaliana*

**Fig. 5:**
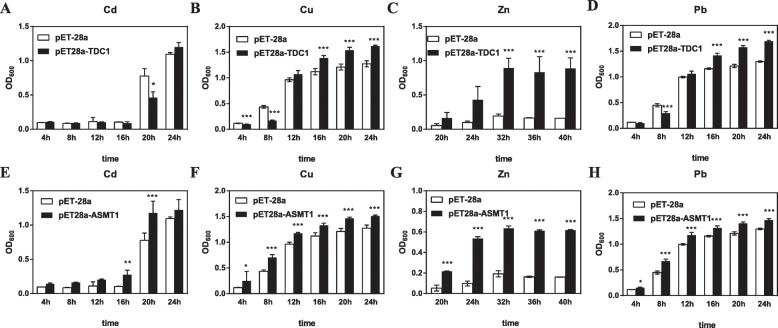
The effects of the overexpression of *EpTDC1* and *EpASMT1* on the optical density at 600 nm (OD_600_) of the transgenic *Escherichia coli* cultures. A total of 1.0 mM isopropyl β-D-thiogalactopyranoside (IPTG) was added to each culture to induce the expression of the recombinant protein. 50.0 mg L^− 1^ Cd^2+^(A, E), 100.0 mg L^− 1^ Pb^2+^(B, F), 70.0 mg L^− 1^ Cu^2+^(C, G), and 100.0 mg L^− 1^ Zn^2+^ (D, H) in cultures expressing *EpTDC1* and *EpASMT1*, respectively. Data are means ± SD (n = 4). Asterisks indicate significant differences (*P* < 0.05*, *P* < 0.01**, *P* < 0.001***) compared to the control according to independent-samples t-test

The expression of melatonin biosynthesis enzyme genes in response to heavy metal-induced stress indicated that these were involved in conferring heavy metal tolerance. We transferred *EpTDC1* and *EpASMT1* into *E. coli* and *A. thaliana* to further investigate the role of melatonin in heavy metal-induced stress tolerance. The number of *E. coli* in liquid culture was measured by optical density at 600 nm (OD_600_), which is a widely used method in bacteria [20]. The OD_600_ of *E. coli* overexpressing *EpTDC1* and *EpASMT1* were significantly enhanced under Cd, Cu, Zn, and Pb stresses (Fig. 5a-h). The results indicate that overexpression of both *EpTDC1* and *EpASMT1* conferred *E. coli* heavy metal-induced stress tolerance.

**Fig. 6:**
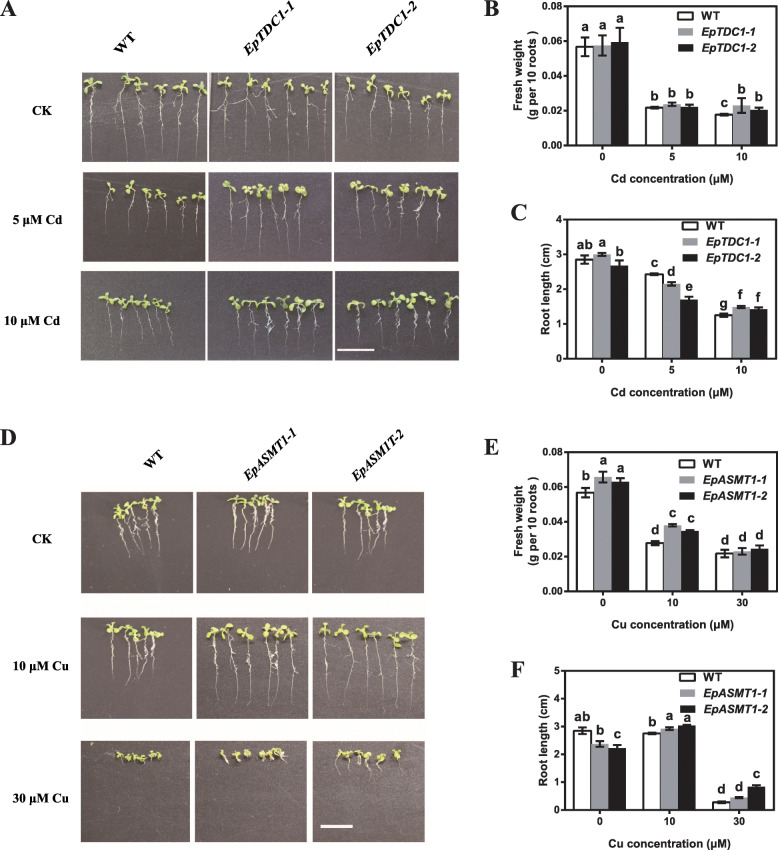
The effects of the overexpression of *EpTDC1* and *EpASMT* on heavy metal-induced tolerance in *Arabidopsis thaliana*. Seeds of the transgenic *EpTDC1* (*EpTDC1–1*/*EpTDC1–2*) and *EpASMT1* (*EpASMT1–1*/*EpASMT1*–2*) A. thaliana* grown on 1/2 strength Murashige-Skoog (MS) medium plates contained 0, 5.0, and 10.0 μM Cd^2+^, and 0, 10.0, and 30.0 μM Cu^2+^ respectively for 10 d. The wild-type (WT) *A. thaliana* was used as control. Photographs showing the growth of *A. thaliana* in Cd and Cu concentrations, respectively (A, D) (scale bars = 1 cm). Meanwhile, the root length (B, E) and fresh weight (C, F) were measured. Data are means ± SD (*n* = 6). Columns with different letters denote significant differences at *P* < 0.05 according to Duncan’s multiple range test

In *A. thaliana*, *EpTDC1–1*/*EpTDC1–2* and *EpASMT1–1*/*EpASMT1–2* transgenic lines were generated to investigate the heavy metal-induced tolerance. The overexpression of *EpTDC1* and *EpASMT1* enhanced the growth of *A. thaliana* in 10.0 μM Cd and Cu concentrations, respectively (Figs. 6a and d). In plants grown in 10.0 μM Cd^2+^, the root length and fresh weight significantly increased by 19.2, 29.6% in *EpTDC1–1* and 14.0, 15.5% in *EpTDC1–2* in comparison with the wild-type (WT) plants (Fig. 6b and c). As in *EpTDC1–1*/ *EpTDC1–2* transgenic plants, the root length, and fresh weight significantly increased by 31.46 and 78.82% in *EpASMT1–1*, and 36.52 and 63.53% in *EpASMT1–2* under 10.0 μM Cu^2+^, respectively (Fig. 6e and f). In this section, it has been indicated that the overexpression of *EpTDC1* and *EpASMT1* relieved heavy metal stresses for transgenic *Arabidopsis*.

### *EpTDC1* and *EpASMT1* decreased Cd accumulation in *A. thaliana* plants*,* especially in roots

**Fig. 7:**
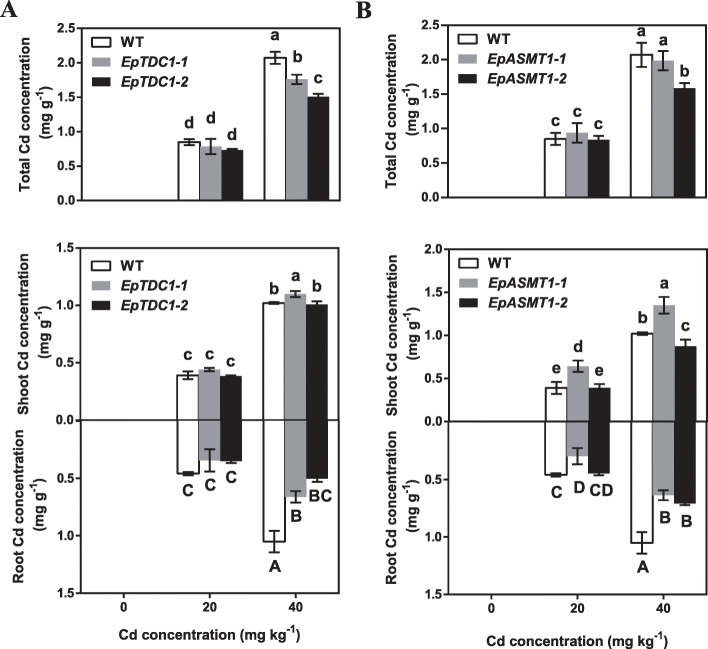
The effects of the overexpression of *EpTDC1* and *EpASMT1* decreased Cd accumulation on Cd concentration in *Arabidopsis thaliana* whole plants, shoots, and roots. The *A. thaliana* seedlings at the four-leaf stage of wild-type (WT), *EpTDC1–1*/*EpTDC1–2*, and *EpASMT1–1*/*EpAMST1–2* were transferred to soil containing 0, 20.0, and 40.0 mg kg^− 1^ Cd^2+^, respectively for 30 d. Data are means ± SD (n = 4). Columns with different letters denote significant differences at *P* < 0.05 according to Duncan’s multiple range test

Our results indicated that exogenous melatonin reduced heavy metal accumulation in *E. pisciphila*. To determine whether *EpTDC1* and *EpASMT1* suppressed the heavy metal accumulation in transgenic *A. thaliana* plants, Cd concentrations were measured. The overexpression of *EpTDC1* and *EpASMT1* showed no significant effect on Cd accumulation in the shoot, root, or the whole plant of *A. thaliana* when grown on 20.0 mg kg^− 1^ Cd. However, a significant decrease in Cd accumulation was observed in the whole plant, especially in roots, when grown on 40.0 mg kg^− 1^ Cd^2+^ (Fig. 7a and b). These data suggested that *EpTDC1* and *EpASMT1* enhanced Cd resistance is associated with decreasing Cd accumulation in the root of *A. thaliana*.

## Discussion

In this study, we elucidated a very important role of melatonin in heavy metal-induced stress tolerance in *E. pisciphila*. High concentrations of heavy metals cause organisms to produce excessive reactive ROS via inhibiting the antioxidant system, disrupting the electron transport chain, and disturbing the metabolism of essential elements [21–23]. Organisms have evolved kinds of mechanisms to relieve oxidative stress caused by heavy metals [24, 25]. As an ancient antioxidant, melatonin plays a key role in lowering oxidative stress [13, 26], thereby potentially conferring heavy metal-induced stress tolerance. We observed that application of exogenous melatonin reduced MDA and OFR concentrations in *E. pisciphila* under Cd, Zn, and Pb stresses (Fig. 1a, c-e, g, and h), suggesting that melatonin may act as an antioxidant to relieve the deleterious impact of heavy metals by alleviating oxidative stress [26]. Melatonin production is promoted to lower heavy metal stress in plants and animals [1, 2].

We also recorded significantly enhanced melatonin accumulation in *E. pisciphila* treated with Cd, Cu, and Zn after 2 d (Fig. 4d). Our study showed that Pb did not promote melatonin production (Fig. 4d), which is consistent with that observed in rice [27], indicating that *E. pisciphila* have distinct stress responses to the different types of heavy metals [28, 29].

Other than directly scavenged ROS, melatonin indirectly activates antioxidant enzymes such as SOD and decreases heavy metal accumulation to relieve heavy metal stress [27, 30]. Melatonin decreased excessive ROS caused by heavy metals in rice, watermelon, and wheat by activating SOD [6, 8, 9]. Our findings also showed that 200.0 μM melatonin significantly increased SOD activity under Zn and Pb stresses (Fig. 2c and d). Hence, melatonin lowered oxidative stress through enhanced the activity of SOD under heavy metal stresses in *E. pisciphila*.

Apart from directly acting as an antioxidant and indirectly activating antioxidant enzymes such as SOD, heavy metal-induced stress can also be alleviated by decreasing heavy metal accumulation [1]. Melatonin application significantly lowered V in *Citrullus lanatus*, and Cd in rice and *Arabidopsis*, respectively [6, 9, 12]. Given that 200.0 μM melatonin significantly reduced the Cd, Zn, and Pb concentrations in *E. pisciphila* (Fig. 2e, g and h), it is likely that melatonin decreased the heavy metal accumulation to enhance heavy metal-induced stress tolerance for this DSE. Our study confirmed that melatonin enhanced heavy metal-induced stress tolerance by increasing SOD activity and reducing heavy metal accumulation in *E. pisciphila* (Fig. 2a-h).

To understand the precise role of melatonin on heavy metal-induced stress tolerance, an investigation of biosynthetic pathways is required [11]. In plants, the first committed step for melatonin biosynthesis is by TDC, and SNAT and ASMT contribute to the two final catalyses [3]. In our previous transcriptome analysis of *E. pisciphila* [19], the expression of three genes annotated as *TDC*, *SNAT*, and *ASMT* was upregulated by Cd. The phylogenetic tree showed that EpTDC1, EpSNAT1, and EpASMT1 differed from that of plants and animals (Fig. 3S1 and S2). Under Cd, Cu, and Zn stresses (2 d), *EpTDC1* and *EpSNAT1* were transcriptionally upregulated with elevated melatonin concentrations (Fig. 4a, b and d). Our findings are in sync with the report on, the upregulation of *TDC* and *SNAT* expression-induced melatonin production with Cd treatment in rice [7]. Our results indicated the essential role of *EpTDC1* and *EpSNAT1* in enhancing melatonin levels for heavy metal-induced stress tolerance in *E. pisciphila.* The reason for the downregulation of *EpASMT1* (Fig. 4c) is not clear, but it may be important for the induction of melatonin production, and further studies are needed to understand this response. Transcript levels, however, are not sufficient to predict protein levels and to explain genotype-phenotype relationships [31]. The report that melatonin production in rice did not consist of the expression of its biosynthesis enzyme genes under Cd stress supports our finding [27].

To further investigate the role of melatonin in heavy metal-induced stress tolerance, we overexpressed *EpTDC1* and *EpASMT1* in *E. coli* (Fig. 5a-h) and *A. thaliana* (Fig. 6a-f)*.* We observed that melatonin helped overcome the growth restriction caused by heavy metals. Consistent with the reports of overexpressed alfalfa *SNAT* in *Arabidopsis* [12], *EpTDC1* and *EpASMT1* decreased Cd concentrations in the whole plant and especially the roots of *A. thaliana* when treated with 40 mg kg^− 1^ Cd^2+^ (Fig. 7a and b). These data indicate that *EpTDC1* and *EpASMT1* played key roles in limiting excessive heavy metal accumulation.

## Conclusions

We demonstrated that melatonin alleviated heavy metal-induced stresses in *E. pisciphila* by directly acting as an antioxidant, indirectly activating SOD, and by reducing heavy metal accumulation. Heavy metal-induced stresses upregulated melatonin biosynthetic enzyme genes (*EpTDC1* and *EpSNAT1*), and promoted melatonin biosynthesis in *E. pisciphila*. We also observed that melatonin helped overcome the growth restriction caused by heavy metals in *E. coli* and *A. thaliana*.

These findings can further be extended to understand the role of melatonin in other DSEs associated with economically important plants growing in soils contaminated with heavy metals. Our study paves a path for research of new strategies in sustainable agricultural practice where plants associated with DSEs can grow in soils contaminated with heavy metals.

## Methods

### Collection of fungal specimens and experimental design

*E. pisciphila* was isolated from the roots of *Arundinella bengalensis* (authenticated by Prof. Shugang Lu from Yunnan University, and the voucher specimen was preserved in Yunnan University), naturally growing in an old mine smelting site in Huize County, Yunnan Province, Southwest China (103°630 E, 26°550 N), and preserved in the Agricultural Culture Collection Center of China (accession number ACCC32496). The fungus was first incubated in Melin-Norkrans (MMN) liquid medium at 28 °C and 180 rpm for 7 d. Then, *E. pisciphila* was pretreated by incubating in the medium supplemented with 0, 50.0, 100.0, or 200.0 μM melatonin for 1 d. Exogenous melatonin dissolved in the medium can be absorbed by organisms as a small amphipathic molecule [27]. *E. pisciphila* was then incubated in the media supplemented with or without Cd^2+^ (111.2 mg L^− 1^), Cu^2+^ (100.0 mg L^− 1^), Zn^2+^ (1010.0 mg L^− 1^), and Pb^2+^ (800.0 mg L^− 1^) for 2 d.

### Determination of malondialdehyde (MDA), oxygen free radical (OFR), and superoxide dismutase (SOD)

A total of 0.5 g fresh hyphae were ground into fine powder with liquid nitrogen in a mortar. The detection of different parameters was performed by Malondialdehyde (MDA) Detection Kit (A003–1-2), Oxygen Free Radical (OFR) Detection Kit (A052–1-1), and Superoxide Dismutase (SOD) Detection Kit (A001–1-2) (Nanjing Jiancheng Bioengineering Institute, China) according to the manufacturer’s instructions.

### Determination of Cd, Cu, Zn, and Pb concentrations in *E. pisciphila*

Hyphae were collected and washed three times with deionized water. Subsequently, samples (0.5 g) were oven-dried at 80 °C, then digested with HClO_4_ and HNO_3_ mixture (1:4 v/v) at 260 °C. The concentrations of Cd, Cu, Zn, and Pb were analyzed using an atomic absorption spectrophotometer (AA240; Shimadzu Co., Kyoto, Japan).

### Quantification of melatonin by high-performance liquid chromatography (HPLC) in *E. pisciphila*

Hyphae (0.1 g) were ground in liquid nitrogen and extracted with 1.0 mL chloroform for 1 h at room temperature before melatonin quantification. Chloroform extracts (200.0 μL) were completely evaporated and dissolved in 0.1 mL 40% (v/v) methanol, and 10.0 μL aliquots were subjected to HPLC using a fluorescence detector system (Waters, Milford, MA, USA). The samples were separated on a 4.6 × 150 mm Sunfire C18 column (Waters, Milford, MA, USA) using the following gradient elution profile: from 42% (v/v) to 50% (v/v) methanol in 0.1% (v/v) formic acid for 27 min, followed by isocratic elution with 50% (v/v) methanol in 0.1% (v/v) formic acid for 18 min at a flow rate of 0.15 mL min^− 1^. Melatonin was detected at 280 nm (excitation) and 348 nm (emission). All measurements were taken in triplicate.

### Identification and phylogenic tree construction of EpTDC1, EpSNAT1, and EpASMT1

Based on the transcriptome database of *E. pisciphila* [19], the nucleic acid sequences of putative *EpTDC1*, *EpSNAT1,* and *EpASMT1* were obtained. The open reading frame (ORF) nucleotide and amino acid sequences of these unigenes were predicted using Open Reading Frame Finder in National Center for Biotechnology Information (NCBI). Then BLAST search was performed using the *EpTDC1*, *EpSNAT1*, and *EpASMT1* amino acid sequences. Phylogenetic trees were generated from various amino acid sequences after alignment with Clustal X (version 1.83), and phylograms were constructed using the Neighbor-Joining algorithm (MEGA 5.0) with branch length.

### Expression analyses of *EpTDC1*, *EpSNAT1*, and *EpASMT1*

Total RNA was extracted from hyphae using the RNAiso Plus 9108 (TaKaRa, Japan) according to the manufacturer’s instructions. The isolated RNA (1.0 μg) was used to synthesize cDNA via the PrimeScriptII 1st Strand cDNA Synthesis Kit 6210A (TaKaRa, Japan). The quantitative real-time PCR (qRT-PCR) analysis was performed with a LightCycler® 480 II Real-Time PCR Detection System (Roche, Basel, Swiss) using SYBR Premix Ex Taq RR820A (TaKaRa, Japan) and *β-tubulin* gene as the internal control. Primers used in this study are listed in Table S2. All the reactions were performed with three biological and technical replicates independently.

### Overexpression of *EpTDC1* and *EpASMT1* in *E. coli* and *A. thaliana*

The ORF sequences of *EpTDC1* and *EpASMT1* were amplified by PCR using the specific primer set containing the restriction endonuclease enzyme sites. For PCR amplification of *EpTDC1*, F: 5′-CGGGATCCATGCTCTGCTTGAGAGGC-3′ (*BamHI* restriction site is underlined) and R: 5′-GGGTTCGAACTTTGGCATGGCCATTC-3′ (*HindIII* restriction site is underlined) as the primer. For *EpASMT*, F: 5′-CGGAATTCATGATGCTAGACAACAAAG-3′ (*EcoRI* restriction site is underlined) and R: 5′- GGGTTCGAACATTAGACCCATCCGTC-3′ (*HindIII* restriction site is underlined) as the primer. The purified PCR products were ligated to the expression vector pET28a (Invitrogen, Carlsbad, CA, USA). Then the final recombinant vectors (pET28a-EpTDC1 and pET28a-EpASMT1) were transformed into *E. coli* BL21(DE3) cells (TaKaRa, Japan).

To obtain transgenic *A. thaliana* plants, *EpTDC1* and *EpASMT1* were amplified by PCR with primers F: 5′-CGGGATCCATGCTCTGCTTGAGAGGC-3′ (*BamHI* restriction site is underlined)/R: 5′-GCTCTAGATTTGGCATGGCCATTC − 3′ (*XbaI* restriction site is underlined) and F: 5′-GGGGTACCATGATGCTAGACAACAAAG-3′ (*KpnI* restriction site is underlined)/R: 5′- GCTCTAGAATTAGACCCATCCGTC-3′ (*XbaI* restriction site is underlined) respectively. The PCR products were inserted into the plant expression vector pCAMBIA1304 (Invitrogen, Carlsbad, CA, USA). The recombinant vectors pCAMBIA1304-35S::*EpTDC1* and pCAMBIA1304-35S::*EpASMT1* were introduced into *Agrobacterium tumefaciens* GV3101 (Invitrogen, Carlsbad, CA, USA) and then transformed into WT *A. thaliana* Columbia-0 (Col-0) (stock CS70000, purchased from the Arabidopsis Biological Resource Center http:// www.arabidopsis.org/abrc) through the floral dip method. The seeds were screened on ½ strength MS supplemented with 25.0 mg L^− 1^ kanamycin in Petri plates. The transgenic *Arabidopsis* plants were confirmed by PCR. The homologous T3 transgenic lines named *EpTDC1–1*/*EpTDC1–2* and *EpASMT1–1*/*EpASMT1–2* were selected for further analysis.

### Growth assessment in transgenic *E. coli* and *A. thaliana* treated with heavy metals

Control (pET28a) and transgenic (pET28a-EpTDC1/pET28a-EpASMT1) *E. coli* BL21(DE3) strains were inoculated into 100.0 mL Luria Broth (LB) liquid media with 1.0 mM isopropyl β-D-thiogalactopyranoside (IPTG). The final concentrations of 50.0 mg L^− 1^ Cd^2+^, 100.0 mg L^− 1^ Zn^2+^, 70.0 mg L^− 1^ Cu^2+^, and 100.0 mg L^− 1^ Pb^2+^ were adjusted in the media separately. The optical density at 600 nm (OD_600_) of the *E. coli* strains was measured at 4 h intervals for different times as described in the corresponding figure legends. Each treatment was conducted independently four times.

Seeds of transgenic *A. thaliana* plants were sown on ½ strength MS medium supplemented with 0, 5.0, and 10.0 μM Cd^2+^ and 0, 10.0, and 30.0 μM Cu^2+^ respectively with the WT *A. thaliana* as the control. After 1 d of stratification at 4 °C, the seedlings were grown for 10 d in a growth chamber at 25 °C, a photosynthetic photon flux density (PPFD) of 100 μmol m ^− 2^ s ^− 1^, and a photoperiod of 16/8 h (day/night). The plants were photographed, and the root length and fresh weight were measured immediately. Each experiment was conducted independently six times.

### Determination of cd accumulation in *A. thaliana*

Seeds of transgenic *A. thaliana* and WT plant were germinated on ½ MS medium in Petri plates. When the second true leaves were fully expanded, seedlings were transferred into the soil fortified with 0, 20.0, 40.0 mg kg^− 1^ Cd^2+^. Plants grown in the soil for 30 d were collected and the Cd concentrations in the whole plant, shoots, and roots were detected by the same method used in *E. pisciphila*. Each experiment was conducted independently four times.

### Statistical analysis

Independent-Samples *t*-test or one-way ANOVA with Duncan’s multiple range test (SPSS 16.0) was used to evaluate the significant differences between means. The data were presented as the mean ± standard deviation (SD). For statistically significant difference, *P*-values < 0.05 were considered statistically significant.

## Supplementary Information

**Additional file 1: Table S1.** Subcellular localization prediction of proteins EpTDC1, EpSNAT1 and EpASMT1. **Table S2.** Primer paris of *Exophiala pisciphila* melatonin synthase genes for qPCR. **Fig. S1.** A phylogenetic tree of serotonin *N*-acetyltransferases (SNATs) based on amino acid sequences. **Fig. S2** A phylogenetic tree of *N*-acetylserotonin *O*-methyltransferases (ASMTs) based on amino acid sequences.

## Data Availability

The datasets used and/or analyzed during the current study are available from the corresponding author on reasonable request.

## Abbreviations

DSE: Dark septate endophyte
ROS: Excess reactive oxygen
RNS: Reactive nitrogen species
TDC: Tryptophan decarboxylase
ASMT: *N*-acetylserotonin *O*-methyltransferase
TPH: Tryptophan hydroxylase
SNAT: *N*-acetyltransferase
Cd: cadmium
Cu: copper
Zn: Zinc
Pb: Plumbum
V: Vanadium
MDA: Malondialdehyde
OFR: Oxygen free radical
SOD: Superoxide dismutase
OD_600_: Optical density at 600 nm
MMN: Melin-Norkrans
ORF: Open reading frame finder
NCBI: National Center for Biotechnology Information
LB: Luria Broth
qRT-PCR: Quantitative real-time PCR
