# Supplementary material for: Melatonin confers heavy metal-induced tolerance by alleviating oxidative stress and reducing the heavy metal accumulation in Exophiala pisciphila, a dark septate endophyte (DSE)
Source: BMC Microbiol. 2021 Feb 5;21:40. doi: 10.1186/s12866-021-02098-1 (PMC7863494; doi:10.1186/s12866-021-02098-1)
Supplement: Supplementary file 1 — Additional file 1: Table S1. Subcellular localization prediction of proteins EpTDC1, EpSNAT1 and EpASMT1. Table S2. Primer paris of Exophiala pisciphila melatonin synthase genes for qPCR. Fig. S1. A phylogenetic tree of serotonin N-acetyltransferases (SNATs) based on amino acid sequences. Fig. S2 A phylogenetic tree of N-acetylserotonin O-methyltransferases (ASMTs) based on amino acid sequences. [file 12866_2021_2098_MOESM1_ESM.docx]

**Table S1.** Subcellular localization prediction of proteins EpTDC1, EpSNAT1 and EpASMT1

| **Protein names** | **Predicated location** |
| --- | --- |
| EpTDC1 | Cytoplasm |
| EpSNAT1 | Cytoplasm |
| EpASMT1 | Nucleus |

**Table S2.** Primer paris of *Exophiala pisciphila* melatonin synthase genes for qPCR

| **Genes** | **Primer paris (5’-3’)** |
| --- | --- |
| *EpTDC1* | Forward: TTCTACCGACCATCAAACCAG  Reverse: GGAATGATCTTGGACCCGATG |
| *EpSNAT1* | Forward: ATGCTGACTGGAAGATTGGG  Reverse: TGCTTGGTTTTCGGGATGTAG |
| *EpASMT1* | Forward: GCGAGTGGAATGATTTGGTTG  Reverse: CTTCGACCTTAGTTTCTCCCTC |


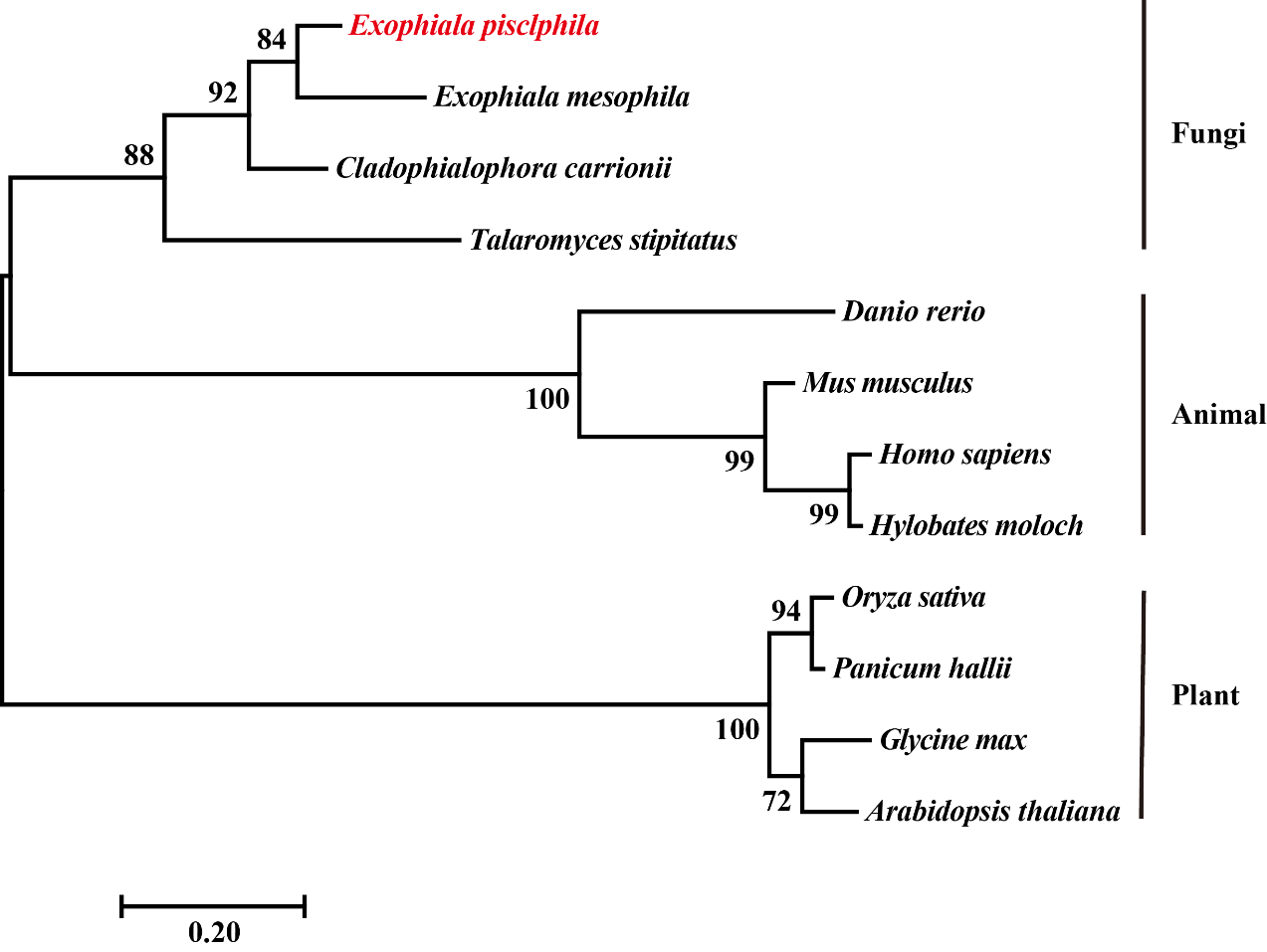


**Fig. S1.** A phylogenetic tree of serotonin *N*-acetyltransferases (SNATs) based on amino acid sequences


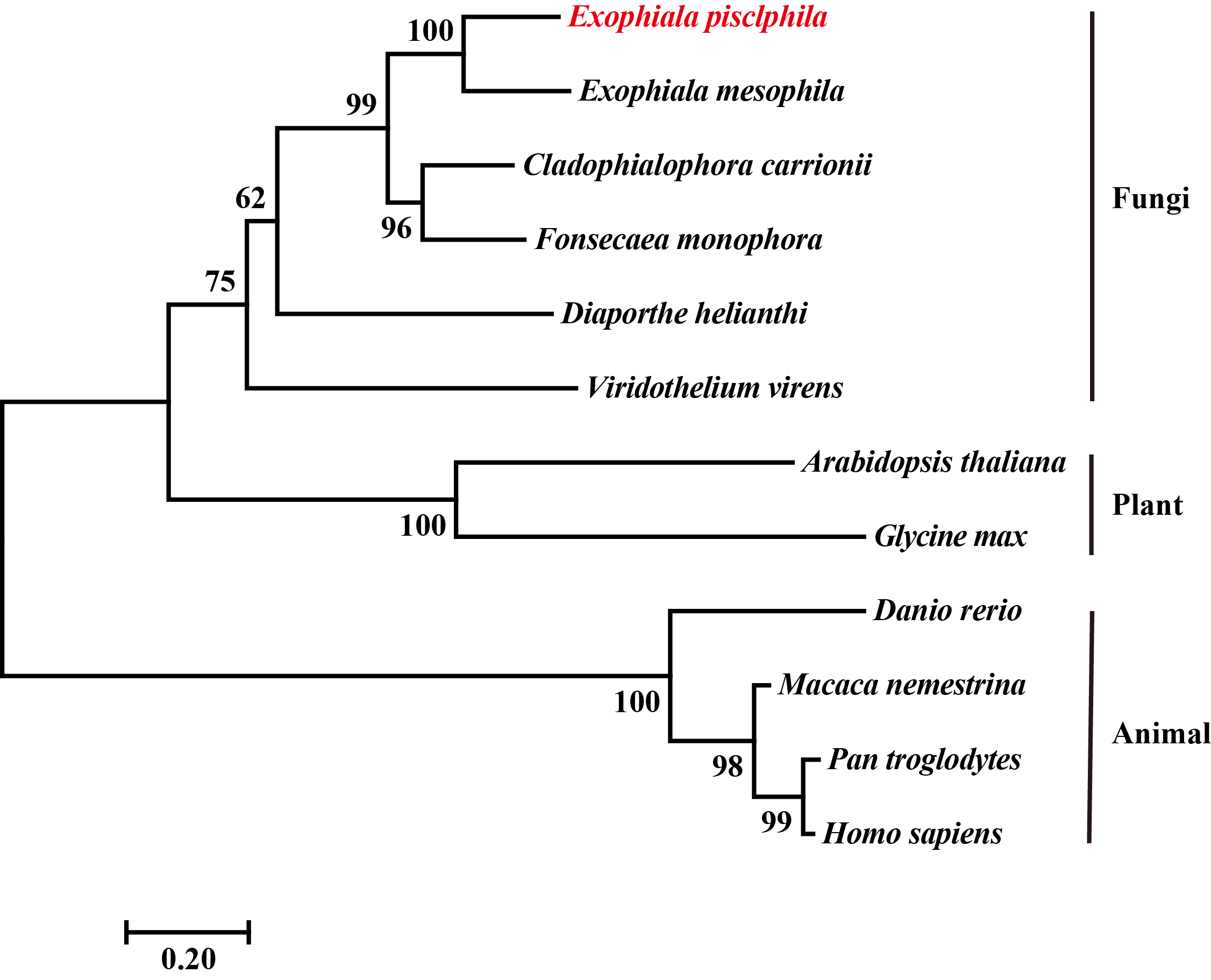


**Fig. S2** A phylogenetic tree of *N*-acetylserotonin *O*-methyltransferases (ASMTs) based on amino acid sequences
